# HAPQ: A Hardware-Aware Pruning and Quantization Pipeline for Event-Based SNN Detection

**DOI:** 10.3390/s26092910

**Published:** 2026-05-06

**Authors:** Zhengyinan Li, Jing Wu

**Affiliations:** 1 School of Automotive Engineering, Wuhan University of Technology, Wuhan 430070, China; qcgcxylzyn@whut.edu.cn; 2 Hubei Provincial Key Laboratory of Modern Auto Parts Technology, Wuhan University of Technology, Wuhan 430070, China

**Keywords:** event-based object detection, hardware-aware co-design, FPGA, structured pruning, membrane potential quantization

## Abstract

Autonomous driving perception demands low latency, high temporal resolution, and stringent hardware efficiency. While event-based spiking neural networks (SNNs) offer bio-inspired sparse computation, their deployment on edge field-programmable gate arrays (FPGAs) is obstructed by irregular execution patterns and temporal state storage overhead. To address this, we propose HAPQ, a unified hardware-aware pruning and quantization pipeline for compact event-based object detection. Starting from an end-to-end adaptive sampling SNN detector (EAS-SNN), HAPQ conducts hardware-aware configuration search within discrete digital signal processor (DSP) and block RAM (BRAM) budgets, applies single-instruction-multiple-data (SIMD)-aligned structured pruning for computational regularity, and jointly quantizes synaptic weights and membrane potentials via a shift-friendly fixed-point recurrence. Evaluation on the Prophesee Gen1 dataset and an FPGA accelerator shows that HAPQ improves detection accuracy from 0.284 to 0.425 in mean average precision ($mAP_{50:95}$) and achieves 0.722 $mAP_{50}$. Hardware implementation reveals a reduction in lookup table (LUT) usage to 1680, complete DSP elimination, and a maximum operating frequency of 920.81 MHz at 0.630 W. These results confirm that effective temporal SNN deployment requires joint optimization of model architecture, state precision, and hardware-aligned workload organization.

## 1. Introduction

Autonomous driving perception must satisfy several constraints simultaneously, including low latency, robustness under fast ego-motion, and stable sensing under abrupt illumination changes. Event cameras are attractive in this setting because they report brightness changes asynchronously, offering substantially finer temporal resolution and wider dynamic range than conventional frame-based sensors in challenging driving scenes [1,2]. However, this sensing advantage can easily be diluted when downstream processing converts events into dense, frame-like representations and applies conventional dense computation [1]. The central question is therefore not only how to sense with events, but also how to preserve their computational advantage throughout the detection pipeline.

This tension is already visible in event-based object detection. Recurrent Vision Transformers (RVTs) [3], Scene Adaptive Sparse Transformer (SAST) [4], Group Event Transformer (GET) [5], and memory-centric asynchronous spatio-temporal networks [6] have all improved benchmark accuracy and clarified how temporal cues in event streams can be aggregated for detection. More recent detectors such as Detecting Every Object from Events [7] and the MoE heat-conduction detector with a new benchmark [8] further illustrate the strong momentum toward higher-performance event-based detection. At the same time, embedded event-based object detection studies [9] make a complementary point: once deployment enters the discussion, the main bottleneck is no longer whether events are informative enough, but whether accurate detectors can meet tight hardware budgets in latency, memory, and power.

Spiking neural networks (SNNs) are a natural candidate for this problem because both event cameras and SNNs operate sparsely over time. Spiking-YOLO first showed that object detection can be formulated in the spiking domain within a familiar detector structure [10]. Trainable Spiking-YOLO then demonstrated that competitive spiking detection requires task-aware training choices rather than a simple direct conversion recipe [11]. More recently, EAS-SNN showed that low-timestep event-based detection can reach competitive accuracy, making it a useful starting point for deployment-oriented design rather than only a proof of concept for SNN detection [12]. Even so, replacing a conventional backbone with an SNN does not by itself solve the deployment problem.

Recent SNN compression studies make this limitation increasingly clear. SpQuant-SNN [13] shows that low-precision membrane-state quantization combined with structured sparsity can improve practical deployability, while Q-SNNs [14] demonstrate that jointly quantizing synaptic weights and membrane potentials is important because temporal state precision directly affects storage and computation. A broader study on pruning and quantization for spiking neural networks [15] similarly suggests that weight compression alone is insufficient once temporal dynamics are involved. On the pruning side, SPEAR [16] connects pruning decisions to Synaptic Operations (SynOps)-constrained execution cost, and Workload-Balanced Pruning [17] shows that nominal sparsity can still degrade effective utilization when temporal or channel-wise imbalance is introduced. Lottery-ticket analyses in SNNs [18,19] further suggest that trainable sparse subnetworks may exist, but trainability does not automatically imply efficient execution on real hardware. Taken together, these studies indicate that three issues repeatedly reappear in deployment: compressing weights alone leaves important costs untouched, irregular sparsity is difficult to exploit efficiently, and persistent temporal states can dominate the hardware budget.

This observation points to a more consequential gap. Event-based SNN detection still lacks a deployment strategy that treats architecture selection, compression, and hardware mapping as one coupled problem. At the optimization level, sparse surrogate-gradient methods [20] and differentiable training formulations for spiking dynamics [21,22] have improved the trainability of temporal SNNs, but they do not directly address deployment-aware compression. In conventional deep networks, integer-arithmetic-only inference [23], learned Step Size Quantization [24], hardware-aware automated quantization [25], and Hessian-aware mixed-precision quantization [26] collectively show that compression becomes markedly more useful once hardware constraints are built into the optimization process itself. What remains missing is an integrated pipeline that brings these ideas together for compact event-driven SNN detectors on FPGA-class platforms.

**Figure 1:**
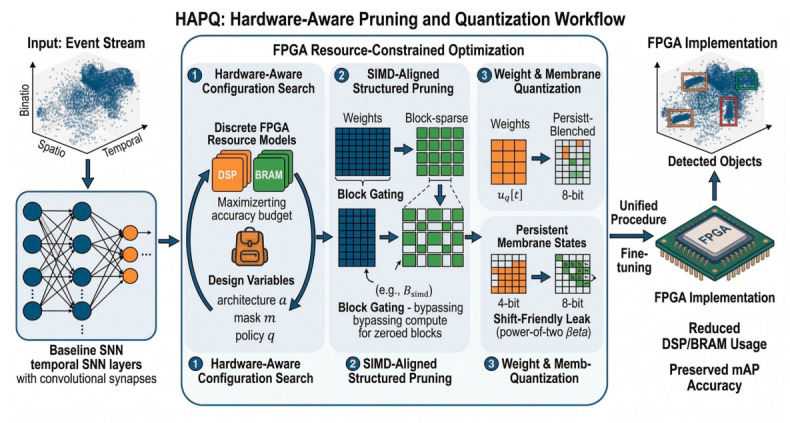
Overview of the proposed HAPQ pipeline.

To address these challenges, this work introduces hardware-aware pruning and quantization (HAPQ), a pipeline for event-based object detection (Figure 1). Using EAS-SNN as the starting point [12], HAPQ combines hardware-aware configuration search, SIMD-aligned structured pruning, and dual quantization of synaptic weights and membrane potentials.

As shown in Figure 1, HAPQ starts from a compact EAS-SNN detector and proceeds through three deployment-oriented stages: hardware-aware configuration search under discrete DSP/BRAM budgets, SIMD-aligned block-wise structured pruning, and dual quantization of synaptic weights and membrane states. The figure summarizes the central co-design logic of this work, namely that temporal state representation and hardware-matched workload organization should be optimized jointly rather than separately.

Our premise is that deployability in temporal SNNs depends on two coupled design decisions: how the temporal state is represented and whether the resulting workload matches the structure of the target hardware. The main contributions of this work are summarized as follows:

- We propose HAPQ, a unified hardware-aware deployment pipeline for event-based SNN detection, jointly performing configuration selection under discrete hardware budgets, structured pruning, and low-bit quantization so that model compactness and implementability are optimized together.
- We introduce two deployment-oriented design choices that specifically target temporal SNN execution on FPGA platforms: SIMD-aligned block-wise structured pruning for regular computation mapping, and shift-friendly quantized membrane recurrence for low-cost state update and storage.
- We validate the proposed framework on the Prophesee Gen1 dataset and an FPGA accelerator implementation. The resulting design improves detection accuracy over a naive fixed deployment while reducing LUT usage and power consumption, eliminating DSP usage, and increasing the maximum operating frequency, thereby confirming the necessity of joint model-hardware co-design for practical edge deployment.

## 2. Related Work

### 2.1. Event-Based Perception and Detection Under Driving Constraints

Event cameras report asynchronous brightness changes with microsecond-level timing and high dynamic range, which makes them well suited to driving scenes dominated by fast ego-motion and abrupt illumination shifts [1,2]. This sensing model changes not only the input modality but also the way temporal information should be represented and processed [1]. However, the efficiency advantage of event sensing becomes fragile when events are densified into frame-like tensors and processed by conventional dense pipelines. This trade-off is clearly reflected in event-based object detection.

Recent event-based detectors follow several distinct architectural directions. RVT [3] uses Recurrent Vision Transformers to model temporal structure over event streams. SAST [4] introduces sparse transformer mechanisms to better adapt computation to event sparsity. GET [5] proposes a Group Event Transformer design that improves temporal aggregation, while the asynchronous spatio-temporal memory network [6] emphasizes memory-centric temporal modeling for continuous event detection. More recent work such as Detecting Every Object from Events [7] and the MoE heat-conduction detector with a new benchmark [8] continues to push detection accuracy upward. At the same time, embedded event-based object detection with SNNs [9] highlights a different issue: as performance improves, computational and memory pressure also increase, so the practical bottleneck increasingly becomes whether these detectors can satisfy embedded latency and power constraints rather than whether events are useful for detection.

### 2.2. Spiking Detectors for Event Streams and the Role of EAS-SNN

SNNs are often viewed as a more natural compute substrate for event streams because sparse spikes and stateful dynamics can, in principle, align neural computation with the event flow. Spiking-YOLO first demonstrated that object detection can be expressed in the spiking domain within a familiar detection template, mainly to explore its energy-efficiency potential [10]. Trainable Spiking-YOLO then clarified an important practical point: competitive spiking detection depends on training-time adaptation and task calibration rather than on a direct conversion formula alone [11]. Building on this line of work, EAS-SNN combines adaptive sampling and event representation with recurrent spiking dynamics in an end-to-end detector, making it a suitable baseline for deployment-oriented design rather than only a concept-level spiking detector [12].

Even with a detection-oriented SNN baseline, deployment cost does not disappear. Once the target platform shifts from GPU evaluation to FPGA-class edge hardware, persistent membrane states, variable spike activity, and irregular workloads remain dominant sources of overhead [9]. This is why event-based SNN detection still cannot be reduced to “replace the backbone with an SNN and then quantize it later.” The deployment problem is structural rather than purely architectural.

### 2.3. Hardware-Aware Deployment: State Precision, Structured Compression, and Accelerator Mapping

Recent SNN compression studies increasingly recognize that compressing synaptic weights alone is insufficient, because membrane potentials and other neuron states are updated at every timestep and can dominate memory traffic. Q-SNNs [14] explicitly quantizes both synaptic weights and membrane potentials, showing across multiple classification and neuromorphic benchmarks that dual quantization can reduce storage and computation while preserving competitive accuracy. SpQuant-SNN [13] moves closer to practical deployment by combining low-precision membrane-state quantization with structured sparsity, and it further extends the evaluation to event-based object detection on Prophesee Gen1 using a Custom-YoloV2-SNN detector. A broader study on pruning and quantization in SNNs [15] reaches a similar conclusion: deployment-oriented compression must consider temporal state variables rather than focusing only on static parameters. These studies are directly relevant here because they establish membrane-state precision as a first-order deployment variable rather than a minor implementation detail.

At the same time, pruning research has moved from parameter removal alone toward workload-aware and hardware-conscious criteria. SPEAR [16] associates pruning with SynOps-constrained search, thereby connecting structural compression to spike-driven execution cost. Workload-Balanced Pruning [17] further shows that sparsity can reduce real utilization when it introduces temporal or channel-wise imbalance, even if the nominal parameter count is lower. Lottery-ticket studies in SNNs [18,19] support the existence of trainable sparse subnetworks, but they also suggest that trainability by itself is not enough to guarantee efficient execution on real hardware. In parallel, optimization-oriented studies such as sparse surrogate gradients [20] and differentiable spike-based training methods [21,22] improve temporal SNN trainability, but they remain largely orthogonal to deployment-aware workload shaping.

From the hardware perspective, conventional deep-learning research has already shown the value of hardware-aware compression. Integer-arithmetic-only inference [23] established the practical benefit of fixed-point deployment. Learned Step Size Quantization [24] improved low-bit training stability. HAQ [25] and HAWQ [26] demonstrated that mixed-precision policies become substantially more effective when hardware properties are incorporated into the optimization process. For hardware mapping more directly, FPGA-oriented pruning for deep-learning accelerators [27] shows that structurally regular compression is more useful than nominal sparsity alone; direct analyses of the hardware impact of quantization and pruning in SNNs [28] confirm that arithmetic savings do not automatically translate into efficient implementation; recent surveys of FPGA-based SNN deployment [29] emphasize the continuing importance of memory movement and state storage; fast FPGA simulation methods [30] address execution efficiency from the algorithmic side; and design-space exploration on digital neuromorphic architectures [31] highlights the need to optimize representation, execution, and hardware constraints jointly. Dedicated neuromorphic platforms, including Loihi [32], Tianjic [33], TrueNorth [34], the million-neuron spiking integrated circuit [35], and SpiNNaker [36], further demonstrate that purpose-built silicon can deliver substantial energy and latency advantages for spike-based workloads, reinforcing why hardware-aware design is not optional but structural. Taken together, these studies suggest that state precision, structured workload shaping, and hardware mapping should be considered simultaneously rather than sequentially.

This distinction is important for fair comparison. Methods such as Q-SNNs [14] and SpQuant-SNN [13] are methodologically close to HAPQ because they address low-precision SNN deployment, but they differ substantially in task definition, detector architecture, benchmark choice, and hardware-validation depth. Q-SNNs [14] mainly establish dual quantization in classification-oriented settings. SpQuant-SNN [13] is closer to our problem because it reaches Prophesee Gen1 detection, but it does not report the same FPGA post-mapping deployment chain used in this work. SPEAR [16] and Workload-Balanced Pruning [17] are highly relevant to structured SNN compression, yet neither serves as a one-to-one baseline for compact event-based object detection with FPGA post-mapping validation. HAPQ therefore addresses a narrower and more deployment-constrained setting: compact event-based object detection on Prophesee Gen1 with an FPGA-oriented co-design loop that jointly considers configuration search, SIMD-aligned structured pruning, membrane-state precision, and hardware mapping.

Table 1 shows that recent SNN compression methods are closely related to HAPQ in terms of methodology but differ substantially in task setting and at the deployment level. Q-SNNs mainly validate dual quantization in classification-oriented benchmarks, whereas SpQuant-SNN is closer to our setting because it reaches Prophesee Gen1 detection. However, these methods do not report the same FPGA post-mapping deployment chain used in this work. Therefore, they are methodologically adjacent, but not one-to-one deployment baselines for HAPQ.

## 3. Methods

We target event-based object detection on edge FPGAs, where the detector has to fit strict DSP and BRAM budgets without collapsing detection accuracy. We start from an end-to-end event-driven detector in the style of EAS-SNN [12] and adapt it with deployment constraints treated as part of the model design rather than an afterthought. HAPQ combines a configuration search evaluated with discrete FPGA cost models, block pruning aligned with the SIMD width exposed by the hardware, and low-precision representations for both synaptic weights and membrane state. This design reflects a practical deployment constraint: sparse or low-bit models are not automatically faster on FPGA. They help only when the induced structure matches the granularity at which the device schedules arithmetic and stores data [13,37].

### 3.1. Baseline Detector, Event Representation, and Temporal State Definition

Let $D=\{(x_{n},y_{n}){\}}_{n=1}^{N}$ denote an event-based detection dataset, where $x_{n}$ is an event stream and $y_{n}$ contains the corresponding bounding boxes and class labels. In contrast to frame-based inputs, event streams can differ sharply in activity level across scenes, so the amount of useful computation also varies from sample to sample. We therefore evaluate the detector with standard metrics such as mean average precision (mAP) and optimize it with a differentiable surrogate objective during training [1].

Our detector is an SNN $f_{\Theta }$ that maps an input event stream to predictions, $\hat{y}=f_{\Theta }(x)$ [12]. For layer l and timestep t∈{1,…,T}, let $s^{\left(l-1\right)}[t]\in \{0,1{\}}^{N_{l-1}}$ denote the input spikes, $u^{\left(l\right)}[t]\in R^{N_{l}}$ the membrane potentials, and $s^{\left(l\right)}[t]\in \{0,1{\}}^{N_{l}}$ the output spikes. We use a discrete-time leaky integrate-and-fire (LIF) neuron with soft reset:

(1) $$\begin{array}{c} u^{\left(l\right)}[t]=\beta^{\left(l\right)}u^{\left(l\right)}[t-1]+I^{\left(l\right)}[t]-\theta^{\left(l\right)}s^{\left(l\right)}[t-1], \end{array}$$

(2) $$\begin{array}{c} s^{\left(l\right)}[t]=H\left(u^{\left(l\right)}[t]-\theta^{\left(l\right)}\right), \end{array}$$

where $\beta^{\left(l\right)}\in (0,1)$ is the leak factor, $\theta^{\left(l\right)}$ is the firing threshold, and H(⋅) is the Heaviside step. For convolutional synapses,

(3) $$\begin{array}{c} I^{\left(l\right)}[t]=W^{\left(l\right)}*s^{\left(l-1\right)}[t], \end{array}$$

where $W^{\left(l\right)}$ is the weight tensor and ∗ denotes convolution.

Because H(⋅) is not differentiable, we train the network with a surrogate gradient approximation commonly used in temporal SNN optimization:

(4) $$\begin{array}{c} \frac{\partial s^{\left(l\right)}[t]}{\partial u^{\left(l\right)}[t]}\approx \phi \left(u^{\left(l\right)}[t]-\theta^{\left(l\right)}\right), \end{array}$$

where ϕ(⋅) is a differentiable proxy [38,39].

During training we optimize the detector with the differentiable loss $L_{det}$, and at test time we report mAP:

(5) $$\begin{array}{c} L_{det}=L_{cls}(\hat{p},p)+\lambda_{box}L_{l_{1}}(\hat{b},b)+\lambda_{iou}L_{iou}(\hat{b},b), \end{array}$$

where $\hat{p}$ and $\hat{b}$ are the predicted class probabilities and bounding boxes. This split is deliberate: deployment quality is judged by mAP, but stable optimization requires a smooth objective that can be differentiated through the temporal SNN.

### 3.2. Hardware-Aware Configuration Search Under FPGA Resource Constraints

- Architecture configuration a (stage-wise channel widths or module choices).
- Structured pruning mask m (block-level activation variables).
- Bitwidth policy $q=(b_{W},b_{U})$ for weights and membrane states.

The resulting network is written as $f_{\Theta }(x;a,m,q)$.

#### 3.2.1. Spike-Rate-Aware Compute

In an SNN, layer cost depends on neural activity rather than layer dimensions alone. To expose that dependence in the hardware model, we define the average spike rate at layer l as

(6) $$\begin{array}{c} \rho^{\left(l\right)}=\frac{1}{TN_{l}}\sum_{t=1}^{T}\sum_{i=1}^{N_{l}}s_{i}^{\left(l\right)}[t]. \end{array}$$

For a convolutional layer, let ${SynOps}_{dense}^{\left(l\right)}$ denote the dense per-step synaptic accumulation count. Under structured pruning with keep ratio $\kappa^{\left(l\right)}(m)\in (0,1]$, we approximate the effective workload by

(7) $$\begin{array}{c} {SynOps}^{\left(l\right)}\approx T\cdot \rho^{\left(l-1\right)}\cdot {SynOps}_{dense}^{\left(l\right)}\cdot \kappa^{\left(l\right)}(m). \end{array}$$

#### 3.2.2. Discrete FPGA Resource Models

We constrain the search with FPGA-visible resources. In particular, mixed precision is only worthwhile when it changes how arithmetic lanes or on-chip memories are packed on the target fabric, so we model those discrete quantization effects explicitly [37,40].

**DSP usage**

Let $\nu_{DSP}(b_{W})$ denote the bitwidth-dependent SIMD packing factor, i.e., the number of MAC lanes that can be packed into one DSP slice. We model the DSP requirement of layer l as

(8) $$\begin{array}{c} R_{DSP}^{\left(l\right)}(a,m,q)=\frac{P_{req}^{\left(l\right)}(a,m)}{\nu_{DSP}(b_{W}^{\left(l\right)})}, \end{array}$$

where $P_{req}^{\left(l\right)}$ is the number of parallel accumulation lanes implied by (a,m).

**BRAM usage**

BRAM cost is discrete and sensitive to width alignment, so we model it as

(9) $$\begin{array}{c} R_{BRAM}(w,d)=\frac{w\cdot d}{C_{BRAM}}\cdot \psi (w), \end{array}$$

where $C_{BRAM}$ is the physical BRAM capacity and ψ(w)≥1 accounts for width-packing penalties. Total BRAM usage is then decomposed as

(10) $$\begin{array}{c} R_{BRAM}=\sum_{l}(R_{BRAM,W}^{\left(l\right)}(b_{W}^{\left(l\right)},a,m)+R_{BRAM,U}^{\left(l\right)}(b_{U}^{\left(l\right)},a,m)). \end{array}$$

The second term is especially important in temporal SNNs because membrane and state buffers persist over time and can become a major efficiency bottleneck if they remain at high precision [14,19].

#### 3.2.3. Search Objective with Hard Resource Budgets

Given these constraints, we optimize for accuracy under fixed resource budgets and leave latency and energy as implementation-time evaluation metrics measured after FPGA deployment. Formally, we solve

(11) $$\underset{a,m,q,\Theta }{\min}L_{det}(\Theta ;a,m,q)+\lambda_{DSP}[R_{DSP}(a,m,q)-\tau_{DSP}{]}_{+}+\lambda_{BRAM}[R_{BRAM}(a,m,q)-\tau_{BRAM}{]}_{+},$$

where $\left[\cdot {]}_{+}=\max(0,\cdot \right)$ and $\tau_{DSP},\tau_{BRAM}$ are the board-specific budgets. The analytic models are therefore used as search-time feasibility proxies: they rank candidates according to hardware alignment during search, while the final LUT, DSP, frequency, and power numbers are measured after implementation [25,41].

#### 3.2.4. Iterative Search Procedure

The search is executed as an iterative evolution process over 19 generations. Each candidate architecture inherits the EAS-SNN detection template [12] but varies stage-wise channel configuration and bitwidth assignment within the search space defined above. At every generation, candidates are first evaluated by validation accuracy and then filtered against DSP and BRAM constraints. Infeasible candidates receive an explicit penalty and are not promoted as parents for the next generation.

**Figure 2:**
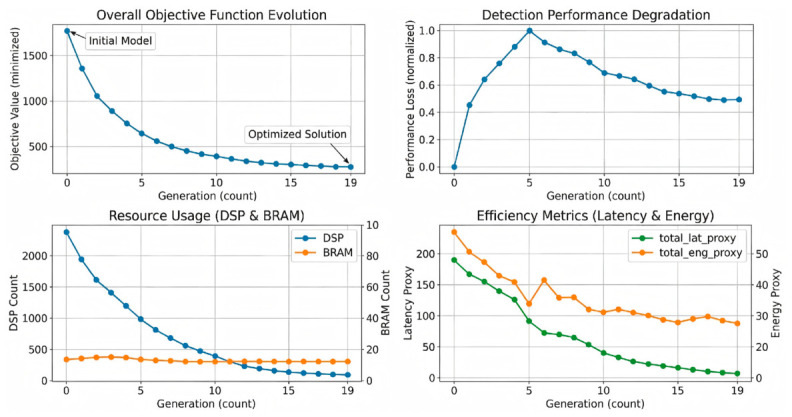
Evolution of spiking neural network detector search under hardware constraints.

The evolution exhibits three practical phases. In the exploration phase (Generations 1–5), the population rapidly removes architectures that violate discrete hardware budgets. In the exploitation phase (Generations 6–10), the search concentrates on high-performing candidates and refines temporal feature-width allocation. In the convergence phase (Generations 11–19), both fitness and validation accuracy plateau, indicating that the search has entered a hardware-feasible neighborhood with stable detection performance. A supplementary visualization of this trajectory is retained in Figure 2.

#### 3.2.5. Fitness Composition and Constraint Handling

The fitness score combines a validation-accuracy term with a resource-overutilization penalty. This design converts hard hardware limits into a searchable objective: architectures that slightly improve mAP but exceed the DSP or BRAM budget are explicitly demoted, whereas architectures that preserve accuracy while respecting discrete packing effects are favored. The selected configuration is then used as the architecture seed for the later pruning and quantization stages.

### 3.3. Block-Wise Structured Pruning

On FPGA, unstructured sparsity rarely delivers the speedup suggested by parameter counts because irregular indexing, control flow, and memory access can erase the arithmetic savings [37]. We therefore prune weights in SIMD-aligned channel blocks so that every retained or removed group matches the execution pattern expected by the hardware [13]. In the implementation used in this work, pruning decisions are applied at a fixed block granularity so that the resulting sparsity can be executed directly rather than only counted statistically.

For each layer l, we partition $W^{\left(l\right)}$ into blocks $g\in G^{\left(l\right)}$ that match the hardware vector width $B_{simd}$. Each block is assigned a binary mask variable $m_{g}^{\left(l\right)}\in \{0,1\}$. The resulting pruned weights are

(12) $$\begin{array}{c} {\tilde{W}}^{\left(l\right)}=W^{\left(l\right)}⊙M^{\left(l\right)}, \end{array}$$

where $M^{\left(l\right)}$ is formed by tiling the block indicators. In deployment terms, this gives us a usable form of block gating: if $m_{g}^{\left(l\right)}=0$, that block is neither fetched nor computed, and we avoid the pointer-heavy irregularity that comes with unstructured sparsity.

**Figure 3:**
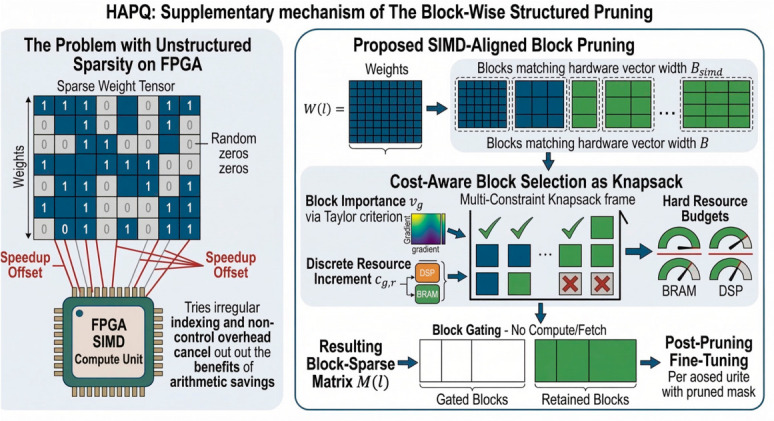
Block-wise structured pruning in HAPQ.

Figure 3 explains why pruning must be aligned with hardware execution granularity. The left panel illustrates the inefficiency of unstructured sparsity on FPGA, where irregular indexing and control overhead weaken the practical benefit of nominal parameter reduction. The right panel shows the proposed SIMD-aligned block pruning strategy, in which block selection is performed under discrete DSP/BRAM constraints and the retained blocks are subsequently fine-tuned to recover detection performance. Instead of removing isolated weights, we group parameters into vector-width-aligned blocks and decide whether each block should be retained as a whole. This avoids irregular fetch and indexing overhead, while allowing the pruning decision to be expressed directly in terms of block masks and resource increments.

(13) $$\underset{m_{g}\in \{0,1\}}{\max}\sum_{g}v_{g}m_{g}\;s.t.\;\sum_{g}c_{g,r}m_{g}\leq \tau_{r},\forall r\in \{DSP,BRAM\},$$

where $v_{g}$ denotes block importance and $c_{g,r}$ is the discrete resource increment incurred when block g is retained.

For block importance, we use the first-order Taylor criterion

(14) $$\begin{array}{c} v_{g}=\left|\left\langle \nabla_{W_{g}}L_{det},\;W_{g}\right\rangle \right|, \end{array}$$

and we compute $c_{g,r}$ from the resource models above. The resulting pruning policy is driven by the marginal DSP and BRAM cost of each block rather than by parameter count alone. Once the mask M is fixed, we fine-tune Θ by minimizing $L_{det}(\Theta ;M)$, following standard post-pruning practice [37].

### 3.4. Quantization of Weights and Membrane Potentials

In our setting, quantization is not only an arithmetic optimization. For SNN deployment, membrane state can consume a meaningful fraction of on-chip storage, so we quantize both weights and membrane potentials using uniform fixed-point formats [19]. Weight precision primarily affects arithmetic cost, whereas membrane-state precision directly affects persistent buffering and recurrent update overhead.

We use a symmetric uniform quantizer:

(15) $$Q_{b}(x;\Delta )=\Delta \cdot clip\left([\frac{x}{\Delta }],\;-2^{b-1},\;2^{b-1}-1\right).$$

This operator is compatible with integer-only inference style quantization [40]. During quantization-aware fine-tuning, we use straight-through estimation (STE), and when low-bit training becomes unstable we also learn the step sizes [24].

**Figure 4:**
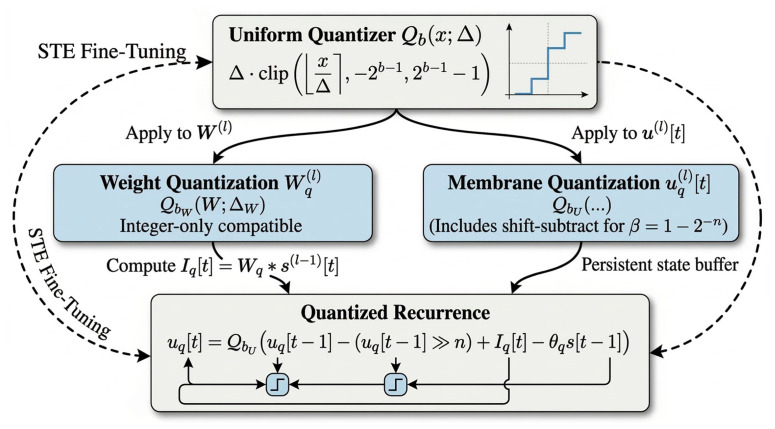
Dual quantization in HAPQ.

The quantizer $Q_{b}(x;\Delta )$ serves as the common operator for both synaptic weights and membrane states. However, in deployment these two variables play different hardware roles: weights primarily determine arithmetic cost, whereas membrane states dominate persistent on-chip storage and recurrent update overhead. Figure 4 summarizes this dual quantization path.

Figure 4 summarizes the dual quantization path used in HAPQ. A common uniform quantizer is applied to both synaptic weights and membrane states, while the membrane branch additionally adopts a shift-friendly recurrent update. This design is important for deployment because membrane-state precision directly affects persistent buffering, data movement, and the hardware cost of timestep updates on FPGA.

To simplify register-transfer level (RTL) implementation, we constrain the leak factors to a power-of-two form:

(16) $$\beta^{\left(l\right)}=1-2^{-n^{\left(l\right)}},n^{\left(l\right)}\in Z_{+},$$

so that the product βu can be implemented as a shift-subtract in fixed-point arithmetic. The resulting quantized recurrence is

(17) $$\begin{array}{c} u_{q}^{\left(l\right)}[t]=Q_{b_{U}^{\left(l\right)}}\left(u_{q}^{\left(l\right)}[t-1]-(u_{q}^{\left(l\right)}[t-1]\gg n^{\left(l\right)})+I_{q}^{\left(l\right)}[t]-\theta_{q}^{\left(l\right)}s^{\left(l\right)}[t-1]\right), \end{array}$$

The bitwidth policy is not fixed purely by intuition. Weight precision is selected to match low-cost fixed-point arithmetic, whereas membrane-state precision is further examined by sensitivity analysis because it changes both numerical fidelity and the effective temporal state space seen during fine-tuning. As shown later in Section 4.4, a moderate membrane bitwidth provides the best trade-off, which supports treating state precision as a searchable design variable rather than a fixed hyperparameter.

### 3.5. Implementation Details and End-to-End Deployment Flow

To make the method reproducible, the main implementation settings are summarized in Table 2. Rather than listing execution steps narratively, this subsection consolidates the concrete configuration values that determine the search space, pruning granularity, quantization-aware fine-tuning, and FPGA deployment flow used in HAPQ.

To further clarify the operational order of the proposed framework, Algorithm 1 summarizes the complete HAPQ procedure from event-stream calibration to FPGA report parsing. The pseudocode complements Table 2 by making the initial inputs, iterative search, structured pruning, dual quantization, and hardware validation stages explicit.
**Algorithm 1:** HAPQ: Hardware-Aware Pruning and Quantization Pipeline**Input:** Event-based training/validation set D;Pretrained compact EAS-SNN detector f(W);Architecture search space A, Bit-width search spaces $B_{W},B_{U}$;SIMD block size g=16, Time steps T=3;Hardware resources budgets $B_{DSP},B_{BRAM}$;Evolutionary generations G=19, QAT epochs E=50.**Output:** Hardware-feasible SNN detector $f_{HAPQ}$, structured mask M, optimal bit-width policy $c^{*}$, Verilog headers, and FPGA hardware reports.**Phase 1: Initialization & Adaptive Representation**1: Convert each event stream in D into the adaptive recurrent SNN representation.2: Initialize population $P_{0}$ sampling from A (stage-wise channel choices) and $\left(B_{W},B_{U}\right)$.**Phase 2: Hardware-Aware Evolutionary Search**3: **FOR** generation k=1 **TO** G **DO**4:          **FOR EACH** candidate $c=(a,b_{W},b_{U})\in P_{k}$ **DO**5:                  Instantiate model $f_{c}$ configured by architecture a and bit-widths $\left(b_{W},b_{U}\right)$.6:                  Estimate layer-wise spike firing rates using a calibration subset.7:                  Compute hardware costs (DSP(c),BRAM(c)) via the discrete FPGA cost model.8:          **IF** $DSP(c)>B_{DSP}$ OR $BRAM(c)>B_{BRAM}$ **THEN**9:                  Assign an over-budget penalty to the candidate’s fitness score.10:        **ELSE**11:                  Fine-tune $f_{c}$ and record the validation mAP as fitness.12:        **END IF**13: **END FOR**14: Select feasible parents and generate $P_{k+1}$ via channel and bit-width mutation/crossover.15: **END FOR**16: Extract the globally optimal feasible configuration $c^{*}=(a^{*},b_{W}^{*},b_{U}^{*})$.**Phase 3: SIMD-Aligned Structured Pruning**17: Partition convolutional weight tensors of $f_{c^{*}}$ into SIMD-aligned channel blocks of size g.18: Calculate block importance via first-order Taylor expansion; map each block to its DSP/BRAM increment.19: Solve the block-retention optimization problem under $\left(B_{DSP},B_{BRAM}\right)$ to generate mask M.20: Apply M to $f_{c^{*}}$ and fine-tune the pruned architecture to recover accuracy.**Phase 4: Dual Quantization-Aware Fine-Tuning (QAT)**21: Quantize synaptic weights and membrane potentials using uniform fixed-point quantizer $Q_{b}$.22: Constrain membrane leak factors to power-of-two values; configure shift-subtract recurrence updates.23: Perform QAT for E epochs using Straight-Through Estimator (STE) and gradient clipping.24: Sweep candidate membrane bit-widths to lock in the optimal accuracy-resource trade-off.**Phase 5: FPGA Deployment & Verification**25: Export pruning mask M, quantization scaling factors, and shift parameters to Verilog headers.26: Execute Vivado batch flow; parse utilization (LUT/FF/DSP), frequency, and power reports.27: **IF** timing closure fails **OR** physical resources exceed bounds **THEN**28:          Fallback to the next-best candidate in $P_{G}$, or tighten search/pruning constraints.29: **END IF**30: **RETURN** $f_{HAPQ}$ and FPGA implementation reports.

This algorithm makes the closed-loop nature of HAPQ explicit: analytic hardware models are used during search and pruning, while the final decision is verified by FPGA synthesis and implementation reports rather than by analytic estimates alone.

## 4. Experiments

### 4.1. Experimental Protocol

We conduct all experiments on Prophesee Gen1 and use the compact EAS-SNN (S) family as the reference platform. This choice is deliberate. On the one hand, recent artificial-neural-network (ANN)-based event detectors such as RVT, SAST, GET, and ASTMNet have pushed the accuracy frontier on Gen1 and related benchmarks. On the other hand, EAS-SNN provides a cleaner starting point for this work because it already couples recurrent spiking representation, low-timestep inference, and a detector-oriented design in a way that is compatible with edge deployment. In other words, it is not only a strong SNN baseline, but also the right bridge between event-based detection and hardware-aware compression.

All internal variants are derived from the same EAS-SNN (S)-based platform with (T = 3), and all post-compression models are trained under the same 50-step fine-tuning schedule. We intentionally keep the fine-tuning budget fixed and short. This makes the comparison harder, but also cleaner: any gain should come from the method itself rather than from extra optimization time.

The external comparison rows are taken from the published Gen1 results reported together with EAS-SNN, while our internal comparison isolates four deployment settings: Full (Fixed), Prune-only (Only structured pruning), Quant-W (Quantization of weight), Quant-W + U (Quantization of weight and membrane potentials), and the full HAPQ pipeline. It separates the effects of structure, quantization, and hardware-aware search instead of hiding them inside a single end result.

### 4.2. Gen1 Comparison: Accuracy Retention After Compression

Table 3 places our results in the broader Gen1 landscape. ANN-based event detectors still define a strong reference line on this benchmark, with RVT and ASTMNet reaching 0.472 and 0.467 *mAP*_50:95_, respectively. Yet the more interesting baseline for this paper is not the ANN ceiling, but the compact SNN regime. In that regime, EAS-SNN (S) already stands out as a meaningful low-timestep detector at 0.372 *mAP*_50:95_.

Our findings make the importance of hardware-aware co-design especially clear. When the compact model is deployed directly in fixed-point form, marked as Full (Fixed), performance drops sharply to 0.284 $mAP_{50:95}$. Compared with the published EAS-SNN (S) reference, this gap is quite revealing. It shows that simply building a compact SNN does not guarantee that it will remain hardware-friendly under fixed-point deployment. The temporal behavior is still sensitive, and a straightforward hardware conversion is not enough.

The Prune-only result offers an even stronger lesson. When structured sparsity is applied without quantization-aware stabilization or architectural adjustment, $mAP_{50:95}$ falls further to 0.265. This aligns closely with recent studies on SNN pruning, which show that structured sparsity is only effective when it preserves temporal workload balance and maintains the synaptic pathways that genuinely carry useful information. Put simply, pruning is important, but pruning alone does not constitute a viable deployment strategy.

The picture begins to improve once quantization is introduced. Quant-W raises $mAP_{50:95}$ to 0.346, recovering a substantial portion of the lost performance. Quant-W+U then pushes it slightly higher to 0.356. This detail is important. In conventional ANN deployment, activation quantization is usually expected. In SNNs, however, the more sensitive quantity is the membrane potential, since it integrates information over time. The fact that low-bit membrane quantization causes no degradation here, and even delivers a modest improvement, supports the conclusions of recent SNN quantization research: when the training process is made aware of the quantizer, compressing state variables can act as a useful regularizer rather than a source of error.

The full HAPQ model reaches 0.722 $mAP_{50}$ and 0.425 $mAP_{50:95}$. Compared with the Full (Fixed) baseline, that represents an absolute gain of 14.1 points in *mAP*_50:95_. More importantly, it does more than simply recover the original compact model’s accuracy. In numerical terms, it even surpasses the published EAS-SNN (S) reference. This provides the clearest experimental evidence that HAPQ is not just compressing a fixed detector. Instead, it actively reshapes the detector so that architecture, sparsity, and precision work together effectively under hardware constraints.

### 4.3. FPGA Deployment: Why the Full Pipeline Matters

The FPGA results in Table 4 explain why the accuracy trends above are not accidental. They also show why the method must be read as a co-design pipeline, not as three unrelated tricks. Prior work on SNN acceleration has repeatedly emphasized that event-driven workloads only become efficient when computation, storage, and scheduling are treated together. Our measurements follow that logic quite closely.

The Full baseline uses 128 DSPs and 2588 LUTs, reaches a maximum frequency of 717.36 MHz, and consumes 0.798 W. That is exactly what we would expect from a compact but still high-precision deployment: the detector functions correctly, but computation and state maintenance still depend on relatively heavy arithmetic and storage resources. The Prune-only configuration reduces DSP and LUT usage, but its maximum operating frequency falls slightly from 717.36 MHz to 702.25 MHz. This is not contradictory. Instead, it shows that even structured sparsity does not automatically lead to better timing closure or improved hardware performance when the time-stepped state path, memory scheduling, and control flow remain mostly unchanged. In streaming SNN accelerators, simply reducing the arithmetic count does not necessarily shorten the critical path. This is exactly why, in the Methods section, we described structured pruning as a workload-aligned constraint rather than a universal shortcut.

The transition from Prune-only to Quant-W shows that weight quantization removes DSP usage entirely, reduces power consumption from 0.757 W to 0.635 W, and increases the maximum frequency to 737.46 MHz. Even so, the timing improvement remains limited compared with the gains observed in later stages. This also aligns with the method analysis: weight quantization reduces arithmetic cost, but it does not yet eliminate the temporal storage bottleneck. That bottleneck becomes clear once membrane quantization is introduced. Moving from Quant-W to Quant-W + U, the Flip-Flop count drops sharply from 74 to 30, and the maximum frequency jumps to 848.90 MHz while power stays low. This is one of the most significant results in the paper. It demonstrates that membrane precision is not just a minor implementation detail. On FPGA, it is a first-order design variable because it directly determines state storage requirements and read–write pressure, exactly as suggested in recent SNN quantization and hardware studies.

The final HAPQ configuration extends this trend further. It reduces LUT usage to 1680, keeps DSP usage at 0, closes timing at an impressive 920.81 MHz, and achieves the lowest power consumption at 0.630 W. Compared with the Full baseline, this corresponds to 35.1% fewer LUTs, complete DSP elimination, a 28.4% higher maximum frequency, and 21.1% lower power consumption. These deployment gains do not come from pruning alone, quantization alone, or NAS alone. They come from aligning all three with the same hardware model.

The operating frequency of 920.81 MHz also has a clear system-level implication beyond timing closure. At this frequency, the accelerator completes one clock cycle in about 1.09 ns, which provides sustained throughput that easily supports the annotation rate of the Prophesee Gen1 dataset, where bounding box labels are available at 1 Hz and event streams are sampled at intervals of up to 200 ms. More importantly, the 28.4% frequency gain over the Full baseline at 717.36 MHz shortens the critical path of the compiled dataflow, which means the same inference task can be completed in less wall-clock time under identical clock-domain constraints. This has practical importance because edge FPGA deployments often run under fixed clock budgets imposed by the larger system board. A higher achievable frequency creates headroom either to run the detector faster or to share the clock domain with other peripherals without introducing timing violations. The frequency improvement is therefore not incidental. It is a direct result of removing DSP-based arithmetic paths and reducing FF state storage, both of which shorten the combinational logic depth along the critical timing path.

### 4.4. Membrane Quantization and Temporal-State Dynamics

Table 5 does not tell a monotonic story. Dropping membrane precision from 16 to 8 bits changes almost nothing—$mAP_{50:95}$ moves from 0.357 to 0.355, essentially noise. Then, 6 bits jumps to 0.395. Then, 4 bits retreats to 0.368. The curve rises and falls, which means something specific is happening: membrane quantization is not simply degrading a signal; it is reshaping the temporal state space through which spikes accumulate, persist, and eventually cross the firing threshold across T = 3 inference steps.

The non-monotonic trend in Table 5 is therefore meaningful. A high bitwidth ($b_{U}\;$= 16) does not produce the best detector, and a very low bitwidth ($b_{U}\;$= 4) does not fully break it. Instead, the strongest result appears at $b_{U}\;$= 6, where the model achieves 0.395 $mAP_{50:95}$ and 0.645 $mAP_{50}$. Because the membrane potential serves as the persistent temporal state of the SNN rather than a simple feed-forward activation, changing its precision affects the resolution of subthreshold accumulation and, as a result, the effective firing behavior. Very high precision preserves many weak state fluctuations, some of which are likely sensitive to noise in event-driven detection, while very low precision makes the accumulation-to-firing transition too coarse. A moderate membrane bitwidth therefore works as a temporal regularizer: it keeps the dominant integration-and-fire behavior intact while filtering out redundant state variation.

This reading is consistent with the compression results reported in Table 3 and Table 4. The Full (Fixed) baseline keeps the compact detector structure, yet its $mAP_{50:95}$ falls to 0.284 after fixed-point deployment, suggesting that the original temporal state path is not well conditioned once precision and hardware constraints are applied. Prune-only lowers it further to 0.265, indicating that structured sparsity by itself may weaken effective spike-propagation pathways when the recurrent state remains insufficiently calibrated. Quant-W restores performance to 0.346 by stabilizing arithmetic precision, while Quant-W + U raises it further to 0.356 because the membrane path itself becomes regularized. HAPQ goes one step beyond this by aligning the architecture, structured sparsity, and state precision with the same hardware objective, which is why the best compressed model is also the one that most effectively preserves temporal discrimination under deployment constraints.

## 5. Discussion

The ablation trajectory in Table 3 and Table 4 makes one thing difficult to ignore: the gains of HAPQ do not stack the way a simple additive story would predict. Pruning alone made detection worse. Weight quantization helped, then plateaued. Only when membrane-state precision was brought into the loop—and only when all three interventions were forced to satisfy the same hardware objective—did performance recover and eventually surpass the pre-compression reference. This is the behavior of a coupled system, not a collection of independent techniques. In temporal SNNs, the recurrent state path is not a passive bystander to compression decisions; it is the variable that either holds the detector together or lets it fall apart, and treating it as an afterthought is precisely what the Prune-only result demonstrates.

The membrane bitwidth study sharpens this reading considerably. A detector running at 16-bit membrane precision does not outperform one at 6-bit—it underperforms, by a margin large enough to matter in practice. The explanation is not counterintuitive once the role of membrane potential is taken seriously: it is a running integral of weighted spike history, updated at every timestep, accumulating subthreshold evidence before committing to a firing decision. High precision preserves every weak fluctuation in that integral, including those driven by the irregular, noise-contaminated bursts that event cameras produce in cluttered scenes. Six bits discard those fluctuations early, before they propagate, acting as a temporal filter rather than a lossy approximation. Four bits overshoot: the quantization grid becomes too coarse to reliably distinguish adjacent accumulation states, and detection degrades. The practical implication is that membrane bitwidth is a design parameter that should be searched, not fixed—which is exactly what HAPQ’s sensitivity sweep formalizes.

The FPGA results support this interpretation. Lower LUT usage, the elimination of DSP consumption, and the increase in maximum operating frequency are not isolated hardware outcomes. Instead, they point to a more regular execution pattern in which state updates, arithmetic mapping, and structured sparsity are better aligned with the target platform. In that sense, HAPQ improves deployment not just by shrinking the model, but by making the detector easier to execute as a temporal hardware workload.

Even with these improvements, an important limitation remains. The current HAPQ framework is optimized for static resource budgets. In other words, the search, pruning, and quantization process assumes a fixed deployment target with fixed DSP and BRAM constraints, along with a fixed compressed configuration after compilation. This is a reasonable assumption for many embedded FPGA applications, but it is less suitable for highly dynamic environments where event-stream density, scene complexity, or runtime workload may vary substantially over time. In such cases, a single fixed compression policy may not always offer the best trade-off among accuracy, latency, and energy efficiency. A configuration that works well for sparse and stable traffic scenes may be less effective when event activity rises sharply in cluttered or high-speed situations.

This limitation also helps define the scope of the present findings. HAPQ should be viewed as a deployment framework for resource-constrained but statically provisioned edge settings, rather than as a runtime-adaptive optimization system. The study shows that strong gains are possible when compression and hardware mapping are co-designed for a fixed target. However, it does not yet address online policy switching, adaptive bitwidth allocation, or dynamic resource reconfiguration in response to changing event statistics. These directions remain important for future work, especially if the goal is to maintain near-optimal efficiency across a wider range of real driving conditions.

Autonomous driving should not be treated as a purely static scenario, but it is also not fully adaptive in the sense of runtime model reconfiguration. From the platform perspective, the current system is deployed on a statically provisioned FPGA target, where available DSP, BRAM, timing budget, and compiled dataflow remain fixed after implementation. In this sense, HAPQ is optimized for a static deployment budget. From the workload perspective, however, autonomous driving is inherently dynamic. Event-stream density, scene complexity, target count, motion intensity, and illumination conditions can all vary substantially across traffic environments, meaning that the detector’s effective computational demand is not constant over time. For that reason, the current framework is best understood as a static deployment optimization method designed for a dynamic sensing scenario.

This distinction matters when interpreting the results. HAPQ shows that meaningful deployment gains can be achieved when architecture configuration, structured pruning, and state precision are co-optimized for a fixed FPGA budget. However, the compressed model remains fixed after compilation, which means it may not always deliver the best balance of accuracy, latency, and energy efficiency under rapidly changing event activity. A configuration that is well suited to sparse highway scenes, for instance, may become suboptimal in cluttered urban traffic or during abrupt illumination shifts. As a result, the present study does not yet solve adaptive deployment under changing workloads. Instead, it establishes a reproducible co-design baseline for statically provisioned edge hardware operating in dynamic real-world conditions. Extending this framework toward runtime-adaptive bitwidth control, workload-aware policy switching, or partial reconfiguration would be a valuable direction for future research.

## 6. Conclusions

This study introduced HAPQ, a hardware-aware pruning and quantization pipeline designed for compact event-based SNN detection on FPGA platforms. Built on top of a compact EAS-SNN detector, HAPQ integrates hardware-aware configuration search, SIMD-aligned structured pruning, and dual quantization of synaptic weights and membrane potentials into a single deployment-focused framework. The main idea behind the method is that efficient temporal SNN deployment depends not only on reducing parameters, but also on jointly optimizing state precision, workload structure, and hardware mapping.

Experiments on Prophesee Gen1 showed that this design strategy works well. Compared with the basic fixed deployment baseline, HAPQ increased detection accuracy from 0.284 to 0.425 in $mAP_{50:95}$ and reached 0.722 in $mAP_{50}$. FPGA implementation results also showed lower LUT usage, zero DSP consumption, reduced power, and a higher maximum operating frequency. Together, these findings suggest that compact event-based SNN detection can be improved significantly when compression is approached as a co-design problem rather than as a simple post-processing step.

The results also highlighted the importance of membrane-state precision in temporal SNN deployment. Sensitivity analysis showed that membrane quantization is more than a storage-saving technique; it is a meaningful design factor that influences both hardware cost and temporal behavior. This supports the broader conclusion that effective deployment requires architecture, sparsity, and state evolution to be shaped together under explicit hardware constraints in temporal SNNs.

In addition, this work offers practical implementation evidence that deployment-oriented co-design can make event-based SNN detection more feasible on resource-limited FPGA platforms. Future research will focus on extending the framework to more adaptive resource settings, stronger event-based detection backbones, and a wider range of hardware targets beyond the current fixed deployment setup.

## Figures and Tables

**Table 1:** Comparability of recent SNN compression and deployment-oriented methods relative to HAPQ.

Method	Task	W/U Quant.	Structured Pruning	Gen1 Detection	FPGA Post-Mapping	Direct Comparability
Q-SNNs [14]	Classification	Yes	No	No	No	Low
SpQuant-SNN [13]	Classification & Detection	Yes	Yes	Yes	No	Moderate
SPEAR [16]	Pruning-oriented SNN compression	No	Yes	No	No	Low
Workload-Balanced Pruning [17]	Sparse SNN pruning	No	Yes	No	No	Low
HAPQ (ours)	Event-based object detection	Yes	Yes	Yes	Yes	High

**Table 2:** Key implementation settings of the HAPQ pipeline.

Category	Item	Setting
Baseline detector	Reference model	Compact EAS-SNN (S) detector
Dataset	Detection benchmark	Prophesee Gen1
Temporal setting	Inference timestep	T=3
Configuration search	Search length	19 generations
Configuration search	Evolution phases	Exploration (1–5), exploitation (6–10), convergence (11–19)
Fine-tuning	Batch size	16
Fine-tuning	quantization-aware training (QAT) learning rate	$2\times 10^{-5}$
Fine-tuning	Fine-tuning epochs	50
Fine-tuning	Gradient clipping	1.0
Structured pruning	Pruning granularity	Block-wise structured pruning
Structured pruning	Block size	16
Structured pruning	DSP budget	dsp_budget=0
Membrane precision	Sensitivity candidates	$b_{U}\in \{16,8,6,4\}$
Membrane precision	Selected bitwidth	$b_{U}=6$
FPGA flow	Hardware deployment path	Verilog parameter export → Vivado batch synthesis → report parsing
Pipeline scripts	Main code modules	tools/train_hapq_event.py; tools/prune.py; tools/run_fpga_flow.py

The table is methodological rather than purely descriptive. It specifies the exact settings under which HAPQ is searched, compressed, fine-tuned, and transferred to the FPGA implementation flow, thereby narrowing the gap between algorithmic formulation and executable deployment. The version of Vivado is v2025.2.

**Table 3:** Performance comparison with the state-of-the-art methods on the Gen1 dataset.

Method	Representation	Backbone	Head	Temporal	Timestep	$mAP_{50}$	$mAP_{50:95}$
RVT [3]	HIST.	Transformer + RNN	YOLOX	Yes	21	-	0.472
SAST [4]	HIST.	Transformer	YOLOX	Yes	21	-	0.482
GET [5]	HIST.	Transformer	YOLOX	Yes	50 ms	-	0.484
ASTMNet [6]	1D TCNN	CNN + RNN	SSD	Yes	3	-	0.467
TR-YOLO [11]	HIST.	SNN	YOLOv3	Yes	3	0.451	-
EAS-SNN (M) °	ARSNN	SNN	YOLOX *	Yes	3	0.731	0.409
EAS-SNN (M) *	ARSNN	SNN	YOLOX *	Yes	3	0.718	0.393
EAS-SNN (M) †	ARSNN	SNN	YOLOX *	Yes	3	0.699	0.375
EAS-SNN (S) *	ARSNN	SNN	YOLOX *	Yes	3	0.687	0.372
EAS-SNN (S) °	ARSNN	SNN	YOLOX *	Yes	3	0.692	0.372
EAS-SNN (S) †	ARSNN	SNN	YOLOX *	Yes	3	0.675	0.354
Full (Fixed, ours)	ARSNN	SNN	YOLOX *	Yes	3	0.456	0.284
Prune-only (ours)	ARSNN	SNN	YOLOX *	Yes	3	0.428	0.265
Quant-W (ours)	ARSNN	SNN	YOLOX *	Yes	3	0.554	0.346
Quant-W + U (ours)	ARSNN	SNN	YOLOX *	Yes	3	0.545	0.356
HAPQ (ours)	ARSNN	SNN	YOLOX *	Yes	3	**0.722**	**0.425**

External baselines are reported in EAS-SNN [12]. All internal variants are built on the EAS-SNN (S)-based platform and use the same 50-step fine-tuning schedule. † denotes a spiking backbone, a spiking feature pyramid, and a spiking detection head. * denotes a spiking backbone, a spiking feature pyramid, and a non-spiking detection head. ° denotes a spiking backbone, a non-spiking feature pyramid, and a non-spiking detection head. ARSNN: adaptive recurrent SNN representation introduced in EAS-SNN [12]. HIST.: event histogram representation. 1D TCNN: one-dimensional temporal convolutional neural network [6].

**Table 4:** Performance compared through deployment on FPGA accelerator implementation.

Mode	LUT	FF	DSP	Freq. (MHz)	Power (W)
Full	2588	74	128	717.36	0.798
Prune-only	2166	74	108	702.25	0.757
Quant-W	2005	74	0	737.46	0.635
Quant-W + U	1981	30	0	848.90	0.632
HAPQ	1680	30	0	920.81	0.630

The measurements are reported after hardware mapping and timing closure.

**Table 5:** Performance of sensitivity of detection accuracy compared by different membrane-potential bitwidths.

$b_{U}$	$mAP_{50:95}$	$mAP_{50}$
16	0.357	0.571
8	0.355	0.587
6	0.395	0.645
4	0.368	0.591

## Data Availability

The code and hardware configuration files supporting the findings of this study, including the HAPQ pipeline, are openly available in GitHub at https://github.com/260653274/HAPQ-SNN-Detection, accessed on 18 March 2026.

## Abbreviations

The following abbreviations are used in this manuscript:

ANN	Artificial Neural Network
BRAM	Block Random Access Memory
DSP	Digital Signal Processor
EAS-SNN	End-to-End Adaptive Sampling and Representation for Event-Based Detection with Recurrent Spiking Neural Networks
FF	Flip-Flop
FPGA	Field-Programmable Gate Array
HAPQ	Hardware-Aware Pruning and Quantization
LIF	Leaky Integrate-and-Fire
LUT	Lookup Table
mAP	Mean Average Precision
NAS	Neural Architecture Search
QAT	Quantization-Aware Training
RTL	Register-Transfer Level
SIMD	Single Instruction Multiple Data
SNN	Spiking Neural Network
STE	Straight-Through Estimation
SynOps	Synaptic Operations
